# A double-blind, randomized, placebo-controlled trial studying the effects of Saccharomyces boulardii on the gastrointestinal tolerability, safety, and pharmacokinetics of miglustat

**DOI:** 10.1186/s13023-015-0297-7

**Published:** 2015-06-19

**Authors:** Tatiana Remenova, Olivier Morand, Dominick Amato, Harbajan Chadha-Boreham, Scott Tsurutani, Thorsten Marquardt

**Affiliations:** Actelion Pharmaceuticals Ltd, Gewerbestrasse 16, Allschwil, 4123 Switzerland; Mount Sinai Hospital, Toronto, Canada; Actelion Pharmaceuticals US, Inc., San Francisco, USA; Münster University Hospital, Münster, Germany

**Keywords:** Type 1 Gaucher disease, Niemann-Pick disease type C, Diarrhea, *S. boulardii*, Miglustat

## Abstract

**Background:**

Gastrointestinal (GI) disturbances such as diarrhea and flatulence are the most frequent adverse effects associated with miglustat therapy in type 1 Gaucher disease (GD1) and Niemann-Pick disease type C (NP-C), and the most common recorded reason for stopping treatment during clinical trials and in clinical practice settings. Miglustat-related GI disturbances are thought to arise from the inhibition of intestinal disaccharidases, mainly sucrase isomaltase. We report the effects of a co-administered dietary probiotic, *S. boulardii*, on the GI tolerability of miglustat in healthy adult subjects.

**Methods:**

In a double-blind, placebo-controlled, two-period, two-treatment cross-over trial, healthy adult male and female subjects were randomly allocated to treatment sequences, A–B and B–A (treatment A - miglustat 100 mg t.i.d. + placebo; treatment B - miglustat 100 mg t.i.d. + *S. boulardii* [500 mg, b.i.d.]). GI tolerability data were collected in patient diaries. The primary endpoint was the total number of ‘diarrhea days’ (≥3 loose stools within a 24-h period meeting Bristol Stool Scores [BSS] 6–7) based on WHO criteria. Secondary endpoints comprised numerous other diarrhea and GI tolerability indices.

**Results:**

Twenty-one subjects received randomized therapy in each treatment sequence (total N = 42), and overall, 37 (88 %) subjects completed the study. The total number of diarrhea days was <1.5 for both treatment sequences, and approximately 60 % of subjects did not experience diarrhea during either treatment period. The mean (SD) number of diarrhea days was lower with miglustat + *S. boulardii* (0.8 [2.4] days) than with miglustat + placebo (1.3 [2.4] days), but the paired treatment difference was not statistically significant (−0.5 [2.4] days; p = 0.159). However, a significant treatment difference (−0.7 [1.9]; p < 0.05) was identified after *post hoc* exclusion of a clear outlier who had a very high number of diarrhea days (n = 13) and inconsistent GI tolerability reporting. The incidence of the GI AEs was higher with miglustat + placebo (82 %) than with miglustat + *S. boulardii* (73 %). There were no between-treatment differences in miglustat pharmacokinetics.

**Conclusions:**

Although the primary endpoint was not met, the results of the *post-hoc* analysis suggest that co-administration of miglustat with *S. boulardii* might improve GI tolerability.

## Background

The reversible glucosylceramide synthase inhibitor, miglustat (Zavesca®; Actelion Pharmaceuticals Ltd, Switzerland), is approved in the EU, the US, and other countries for the treatment of adult patients with mild or moderate type 1 Gaucher disease (GD1) for whom enzyme replacement therapy (ERT) is either unsuitable or not a therapeutic option [1, 2]. Miglustat is also indicated for the treatment of progressive neurological manifestations in adult and pediatric patients with Niemann-Pick type C disease (NP-C) in the EU, Japan, and other countries [2].

Safety and tolerability monitoring during clinical trials in both GD1 [3–6] and NP-C [7–10] has consistently shown gastrointestinal (GI) disturbances such as diarrhea, flatulence, abdominal discomfort, nausea and vomiting as the most frequent adverse effects associated with miglustat therapy. These GI adverse effects occurred primarily during the first 6 months of treatment, and were observed in 80 % of patients during the first 6 months compared with 50–60 % thereafter [2], and have been reported as the most common reason for miglustat discontinuation [11, 12]. GI tolerability is therefore an important matter as it can have an impact on treatment compliance (particularly among young patients with NP-C), thereby reducing treatment benefits.

GI disturbances during miglustat treatment are thought to arise from the inhibition of intestinal disaccharidases, mainly sucrase isomaltase [11, 13–15]. Miglustat (1,5 (butylimino)-1,5-dideoxy-D-glucitol) – a glucose analog – inhibits the human disaccharidase, sucrase, which cleaves sucrose at the O-C (glucose) bond [14, 16, 17]. Furthermore, relatively little inhibition of intestinal lactase, and down-regulation of sucrase expression in gastrointestinal epithelium have been observed [11, 13–15]. Together, these effects lead to maldigestion of sucrose [11], preventing the release of the monosaccharides, glucose and fructose, for further intestinal absorption. Undigested sucrose that is not absorbed subsequently causes osmotic diarrhea [11, 18]. Bacterial fermentation of unabsorbed material can also lead to flatulence and abdominal distension/discomfort [18].

Adaptations within the GI system, such as an enhanced capacity for fermentation in the cecum via adaptation of the microflora, are considered to account for partial resolution of GI disturbances in miglustat-treated patients over time. Additionally, anti-propulsive medications (e.g., loperamide) can help to ameliorate GI disturbances such as diarrhea [3, 18–22]. Dietary modifications at or before initiation of miglustat therapy (e.g., adoption of a low-carbohydrate diet, alongside changes to maintain adequate caloric intake) have also been shown to have some positive impact on GI tolerability [11, 23], and a gradual increase in dose up to the full dose during the first 3 weeks of therapy has been found to be helpful (Amato D, personal communication).

Other methods to improve the GI tolerability of miglustat have been suggested, but as yet there are few published data to support their use. The probiotic delivery of additional sucrase activity to the lower intestine during either initial or ongoing miglustat therapy has been suggested as a way to enhance GI adaptation. A purified formulation of the saccharomyces cerevisiae-derived invertase, sacrosidase (Sucraid®), was shown to be beneficial in patients with congenital sucrase-isomaltase deficiency – a condition where patients experience GI symptoms that are analogous to those observed in miglustat-treated patients [24, 25] – and was also successful in alleviating diarrhea in a pediatric NP-C patient (Marquardt T, personal communication). Administration of lyophilized *S. cerevisiae* (Baker‘s yeast) has also been reported to improve clinical symptoms in children with congenital sucrase-isomaltase deficiency [26]. A Phase 1 clinical study with miglustat showed a positive effect on treatment-related diarrhea after co-administration with Baker‘s yeast extract (*Saccharomyces cerevisiae*; data on file at Actelion).

There is anecdotal evidence of favorable effects from the co-administration of miglustat with *Saccharomyces boulardii* probiotics (Burlina A, personal communication). *S. boulardii* produces a high amount of invertase [27, 28], and may be a good candidate because yeast-secreted sucrase cleaves sucrose at the O-C (fructose) bond [17] and is not expected to be inhibited by miglustat, which is a glucose analog. The probiotic formulation, Florastor® (syn. HANSEN CBS5926; Biocodex, France) contains lyophilized cells of the *S. boulardii* CNCM I-745 yeast strain, and is indicated in both adults and children for the reduction of diarrhea from a number of causes (e.g., recurrent C*lostridium difficile*-associated diarrhea, GI relapses of Crohn’s disease or ulcerative colitis). We report findings from a clinical study that assessed the effect of this widely available probiotic formulation on the GI tolerability profile of miglustat in healthy subjects.

## Methods

### Study design and subjects

This was a randomized, double-blind, placebo-controlled, two-period, two-treatment cross-over Phase 1 trial conducted at the Phase 1 unit at BioPharma Services Inc (Toronto, Canada) in healthy adult subjects between 25 February and 7 June, 2013. The primary objective was to assess whether co-administration of *S. boulardii* decreases the total number of days of diarrhea in healthy subjects receiving miglustat. The safety of co-administration of *S. boulardii* with miglustat, and any effect of *S. boulardii* co-administration on miglustat steady-state pharmacokinetics (PK) were also evaluated.

**Fig. 1:**
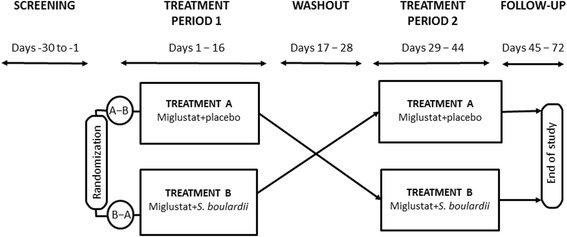
Study design. Subjects received *S. boulardii* or placebo alone (i.e., without miglustat) during the first 2 days of each period

The study comprised a screening period, a two-treatment cross-over period, and a serious adverse event (SAE) follow-up period. Healthy subjects were enrolled and randomly allocated to two different treatment sequences, A-B and B-A, where treatment A was miglustat + placebo and treatment B was miglustat + *S. boulardii*. Treatment allocation was based on a computer-generated, randomization schedule prepared by Clinipace Worldwide. Each treatment period consisted of 14 days of miglustat treatment, with initiation of *S. boulardii*/placebo 2 days before miglustat. The two treatment periods were separated by a 10- to 14-day treatment-free washout (Fig. 1).

### Ethical considerations

The study protocol and all associated materials were reviewed and approved by an independent ethics committee and the study was conducted in full compliance with the Declaration of Helsinki, International Committee on Harmonization guidelines for Good Clinical Practice (GCP), and the US Code of Federal Regulations. All subjects provided written informed consent in line with GCP and local requirements before participation.

### Subjects and treatment

Healthy male and female subjects aged 18–55 years, and with a BMI of 18.5–30.0 kg/m^2^ were included. Women were required not to be lactating or pregnant, and both men and women were required to be using reliable contraception. Study subjects were required not to have any clinically significant findings on the physical examination, laboratory assessment, electrocardiogram (ECG) and vital signs at screening.

Subjects with a known history of yeast allergy, unplanned weight loss of ≥5 % body weight within 1 month or ≥10 % within 6 months of study start, and those who received anti-diarrheal medications (e.g., loperamide), oral probiotic supplements or *S. boulardii* within 30 days, or anti-fungal medications or antibiotics within 8 weeks of study start were excluded from the study. Subjects on any specialized diet were also excluded, as were those with a history of lactose intolerance, irritable bowel syndrome, inflammatory bowel disease (IBD), or other diseases resulting in frequent or severe diarrhea, or a history of GI distress including diarrhea (more than two loose stools per day for ≥5 days) within 30 days of study start.

Doses of miglustat and *S. boulardii* were based on manufacturers’ instructions (Florastor® Biocodex). Miglustat was administered at a dose of 300 mg/day (100 mg t.i.d.) at consistent regular intervals during the day, at least 1 h prior to or after meals (e.g., morning, afternoon, and evening) [2]. *S. boulardii* was taken at doses of 1000 mg/day (500 mg, b.i.d.) with the morning and evening miglustat dose. *S. boulardii* placebo capsules contained only excipients, and were identical in appearance to active *S. boulardii* capsules; both were provided by Biocodex (France).

Compliance with study treatment was monitored based on pill count at each study visit. Subjects had to discontinue if they took <80 % of supplied study medication as per medication reconciliation at the end of each study visit.

Probiotics, other investigational drugs, and any other over-the-counter medications (including homeopathic preparations, herbal medicines, vitamins and minerals) were not allowed during the study. Anti-diarrheal medications were also not allowed unless directed by the principal investigator due to an adverse event.

### Assessments

GI tolerability data were derived from information collated from diaries that subjects filled out daily during 3 days before study drug initiation and on each day during the study treatment periods (i,e., Days 1–16 and Days 29–44). Items addressed in the diaries comprised information on the number of stools, consistency of stools and the presence/absence of several categories of GI distress. Diarrhea was assessed based on WHO criteria [29], the Bristol Stool Score (BSS; a seven-point scale rating stools from 1 [hard stools] to 7 [entirely liquid]) [30] and the Patient-Reported Outcomes version of the Common Terminology Criteria for Adverse Events (PRO-CTCAE).

The primary study endpoint was the total number of ‘diarrhea days’, with one ‘diarrhea day’ defined as ≥3 loose stools within a 24-h period (WHO criteria) that matched BSS diarrhea criteria of 6–7 (based on subject diary information). Secondary endpoints included the number of ‘consecutive diarrhea days’, the frequency and maximum severity of reported GI events (diarrhea, nausea, bloating, belching, flatulence, indigestion/upset stomach, and/or abdominal pain rated on a 5-point scale ranging from 0 [none] to 4 [very severe] inclusive), the mean daily interference score for subjects with GI events (an indication of the extent to which GI symptoms interfered with subjects’ daily activities based on a 5-point scale graded from 0 [not at all] to 4 [very much]), mean daily BSS, the frequency of premature treatment discontinuations related to GI AEs, and the proportion of subjects unable to maintain their usual diet.

### Pharmacokinetics

Miglustat plasma concentrations were measured by Swiss BioAnalytics AG (Switzerland) according to a validated liquid chromatography-tandem mass spectrometry (LC-MS/MS) assay using blood samples taken on the last day of each study treatment period, just before the next miglustat dose (i.e., Days 16 and 46). Miglustat peak concentration (C_max_) and T_max_ were obtained directly from miglustat concentration–time profiles. The area under the concentration–time curve at defined time ‘τ’ (AUC_τ_) was calculated according to the linear trapezoidal rule.

### Safety and tolerability

Safety and tolerability were evaluated based on adverse events (AEs), serious adverse events (SAEs), physical examinations, vital signs, routine hematological, biochemical and urinalysis laboratory assessments, and body weight.

### Data analysis

All randomized subjects in this study took their first dose of study medication. The ‘all randomized’ set was therefore equivalent to the ‘all-treated’ set, and was used to evaluate subject disposition, baseline characteristics, previous and concomitant therapies, treatment exposure, and safety endpoints. The per-protocol (PP) set comprised all subjects from the all-treated set who had completed both treatment Periods 1 and 2 without any major protocol violations. The PP set was used for analysis of the primary endpoint, as well as secondary GI tolerability endpoints. No data imputation was applied to missing study data.

A sample size of 34 subjects was required to provide 80 % power to detect a paired mean difference of 4.0 diarrhea days with an estimated SD of 8.0 diarrhea days at the two-sided significance level of α = 0.05. An assumed 20 % dropout increased the total sample size to 42 subjects. The null hypothesis of no difference between the miglustat + *S. boulardii* and miglustat + placebo treatments was tested against the two-sided alternative hypothesis that it was not equal to zero. The parametric paired *t*-test was used for hypothesis testing if the assumption of normal distribution was met. If not, then the non-parametric Wilcoxon paired signed rank test was used. In addition, *post hoc* sensitivity analyses of the primary endpoint based on the PP set were conducted that excluded an outlier subject, and other continuous GI endpoints were compared between treatments in a similar manner to the primary endpoint using a two-sided paired *t*-test or Wilcoxon paired signed rank test.

Pharmacokinetics variables were estimated using non-compartmental methods based on the PK population, which comprised all treated subjects with completed PK profiles on each end of treatment (EOT) study visit during Periods 1 and 2. The effect of *S. boulardii* on miglustat pharmacokinetics at the end of Period 1 versus Period 2 was assessed by comparing individual and mean plasma miglustat concentration–time profiles and AUC_τ_, C_max_, and T_max_.

## Results

### Subjects and treatment

**Fig. 2:**
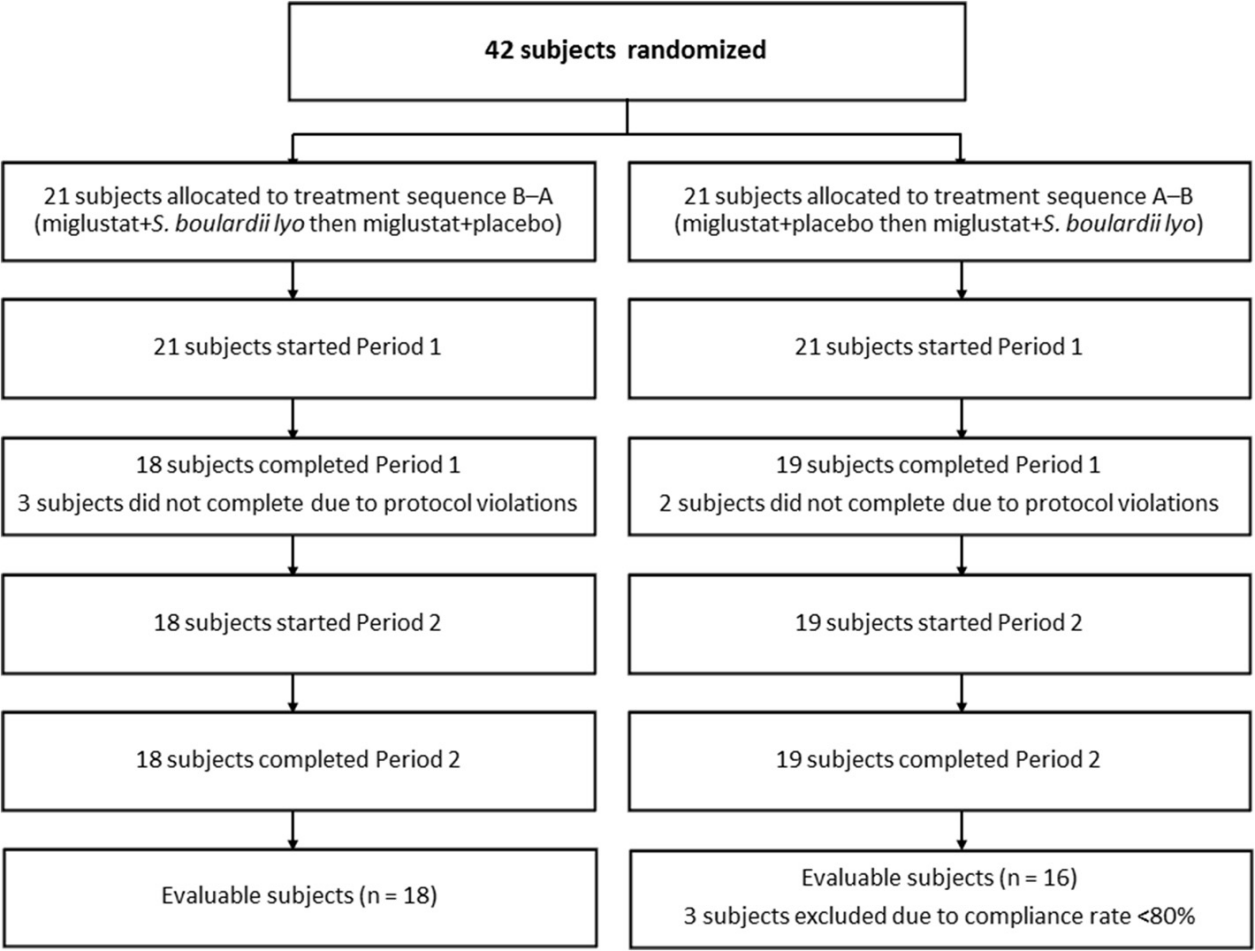
Subject disposition. *All-treated set, *N = 42; PP set, N =* 34. Five patients discontinued prematurely and three subjects had a compliance rate <80 *%*

Subject demographics in the all-treated population are summarized in Table 1, and subject disposition is illustrated in Fig. 2. A total of 42 subjects were screened and enrolled (the all-randomized set that equals the all-treated set), and 21 were randomized to each treatment sequence (i.e., miglustat + *S. boulardii* then miglustat + placebo [n = 21], and miglustat + placebo then miglustat + *S. boulardii* [n = 21]). Thirty-seven subjects (88 %) completed the study, and there were 34 (81 %) evaluable subjects in each of the treatment periods (i.e., N = 34 for the PP population). Eight subjects (19 %) were excluded from the all-treated set due to premature discontinuation in treatment Period 1 (n = 5) or due to dosing compliance rate <80 % (n = 3).

**Table 1:** Baseline characteristics (all-randomized set)

Characteristic	Miglustat + *S. boulardii* then miglustat + placebo	Miglustat + placebo then miglustat + *S. boulardii*	All subjects
	(*n* = 21)	(*n* = 21)	(*N* = 42)
Age, mean (SD)	39.6 (8.9)	35.8 (10.8)	37.7 (10.0)
Gender, *n* (%) male	11 (52)	8 (38)	19 (45)
Race, *n* (%):			
Caucasian	12 (57)	8 (38)	20 (48)
Black	4 (19)	11 (52)	15 (36)
Asian	5 (24)	2 (10)	7 (17)
Mean weight, kg	72.7	71.7	72.2
BMI, mean (SD) kg/m^2^	25.5 (2.9)	25.4 (3.5)	25.4 (3.2)

Subjects were well matched across both treatment sequence groups in terms of age, gender and BMI, and subject characteristics were similar between the all-treated and PP sets. Overall, mean rates of compliance with study treatment throughout the study periods and treatments were >90 % of all subjects.

### Primary endpoint

**Fig. 3:**
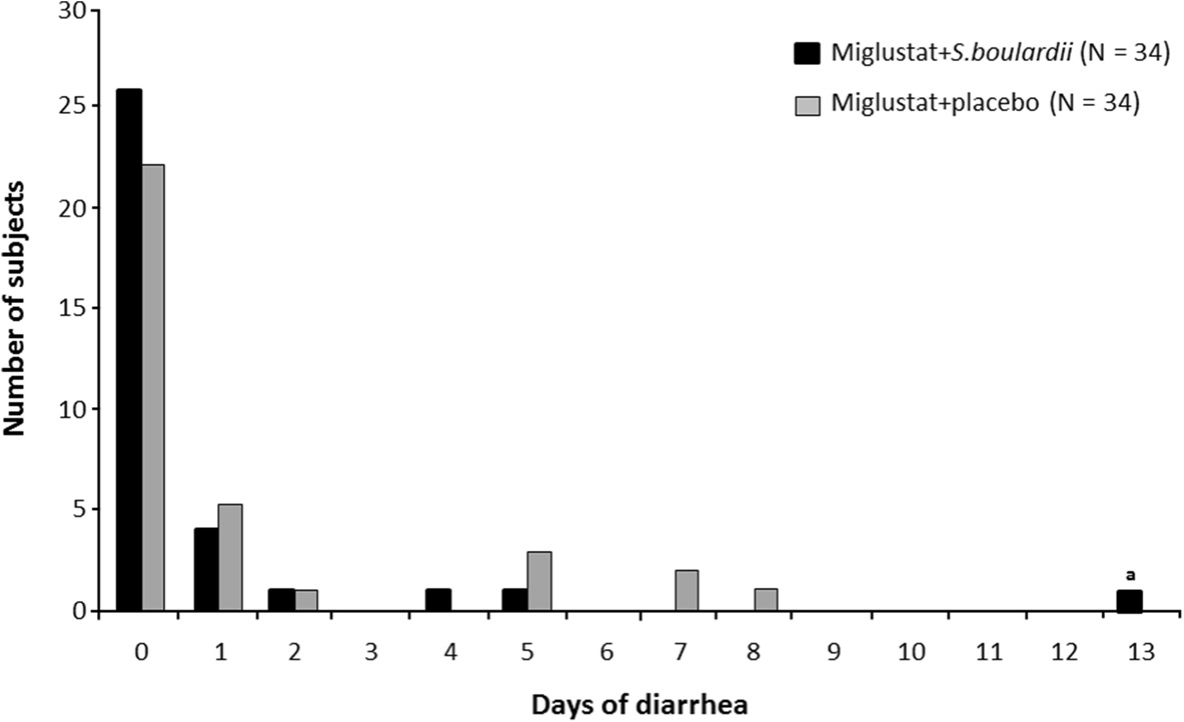
Total number of diarrhea days (PP set). ^a^Outlying subject

The total number of diarrhea days was not normally distributed (Fig. 3), as confirmed based on statistical validity checking based on Shapiro-Wilk (p = 0.0001) and Anderson-Darling (p = 0.0050) criteria. Overall, the total number of diarrhea days defined as per the primary study endpoint was low; approximately 60 % of subjects did not experience diarrhea during either treatment period.

The overall mean number of diarrhea days was <1.5 days for both treatment sequences and across both treatment periods. The mean ± SD number of diarrhea days was lower with miglustat + *S. boulardii* (0.8 ± 2.4 days; range 0–13) than with miglustat + placebo (1.3 ± 2.4 days; range 0–8), but the paired treatment difference in the overall PP analysis (−0.5 ± 2.4 days) was not statistically significant (p = 0.159; Wilcoxon paired signed rank test) (Table 2). Sensitivity analysis based on the frequency of diarrhea days in the all-treated set yielded similar findings (p = 0.1631; miglustat + *S. boulardii* versus miglustat + placebo based on two-sided paired *t*-test).

**Table 2:** Primary endpoint: total number of diarrhea days (PP set)

Total number of diarrhea days	Miglustat + *S. boulardii*	Miglustat + placebo	Paired difference	P-value
	(*n* = 34)	(*n* = 34)	(*n* = 34)	
Mean (SD)	0.8 (2.4)	1.3 (2.4)	−0.5 (2.4)	0.159*^†^
Range (min, max)	0, 13	0, 8	−7, 8	–
95 % CI of mean	0, 1.67	0.46, 2.13	−1.31, 0.37	–
Median	0	0	0	–
Q1, Q3	(0, 0)	(0, 1)	(−1, 0)	–
95 % CI of median	0, 0	0, 1.00	0, 0	–

*Based on paired *t* test; ^†^p = 0.042 based on Wilcoxon paired signed rank test after one outlying subject was excluded during the post-hoc sensitivity analysis

A very high number of diarrhea days (n = 13) was observed in one female subject who was randomized to receive miglustat + placebo followed by miglustat + *S. boulardii*. This subject was considered as an outlier due to her recorded symptoms. She had: 1) WHO-defined diarrhea of unknown cause during the initial 2 run-in days with *S. boulardii* before miglustat initiation; 2) a disproportionately high number of diarrhea days compared with other patients; 3) inconsistencies in her diary entries regarding diarrhea compared with diarrhea judged according to the primary endpoint, which led to contradictory quantitative and qualitative assessments of the number of diarrhea days. An exploratory *post hoc* primary analysis was therefore performed that excluded this outlier subject. Based on the primary endpoint this *post hoc* analysis identified a significantly lower total number of diarrhea days with miglustat + *S. boulardii* (0.5 ± 1.2 days; range 0–5) versus miglustat + placebo (1.2 ± 2.3 days; range 0–8), providing a paired treatment difference of −0.7 ± 1.9 days (p < 0.05; Wilcoxon paired signed rank test).

### Secondary and exploratory endpoint analyses

Secondary efficacy analysis showed that the mean number of consecutive diarrhea days (defined as ≥3 loose stools meeting a BSS score of 6 or 7 within consecutive 24-h periods) was generally low. The mean (SD) numbers of consecutive diarrhea days were 0.5 (1.48) days with miglustat + *S. boulardii* and 0.9 (1.58) days with miglustat + placebo (p = 0.2189 based on Wilcoxon paired signed rank test for miglustat + *S. boulardii* versus miglustat + placebo).

Abdominal bloating and flatulence (mostly mild or moderate) were the most frequent GI events recorded in subject diaries; diarrhea was the next most frequent (Table 3). Most subjects recorded interference scores of 0 (not at all) and 1 (a little bit) based on PRO-CTCAE for all GI events assessed.

**Table 3:** Most frequently reported subject-reported outcomes* of GI events (all-treated set)

	Miglustat + *S. boulardii*	Miglustat + placebo
	(*n* = 40)^†^	(*n* = 39)^‡^
Flatulence		
0 (no event)	13 (33)	12 (31)
1 (mild)	8 (20)	13 (33)
2 (moderate)	11 (28)	6 (15)
3 (severe)	3 (8)	6 (15)
4 (very severe)	4 (10)	2 (5)
Bloating		
0 (no event)	17 (43)	14 (36)
1 (mild)	9 (23)	10 (26)
2 (moderate)	5 (13)	11 (28)
3 (severe)	7 (18)	4 (10)
4 (very severe)	1 (3)	0 (0)
Diarrhea		
0 (no event)	25 (63)	22 (56)
1 (mild)	7 (18)	7 (18)
2 (moderate)	4 (10)	6 (15)
3 (severe)	3 (8)	3 (8)
4 (very severe)	0 (0)	1 (3)

*Only the three most frequently reported GI events are included

^†^Miglustat + *S. boulardii* n = 40 (21 in Period 1 + 19 in Period 2);

^‡^Miglustat + placebo n = 39 (21 in Period 1 + 18 in Period 2)

Overall, the mean (SD) BSS for Day 1 of the study was 3.7 (1.2) for the miglustat + *S. boulardii* treatment group and 3.6 (1.1) for the miglustat + placebo treatment group, but by Day 16 of treatment period 1 the mean (SD) BSS was 4.3 (0.7) and 5.3 (1.0) in these treatment groups, respectively. There were no premature discontinuations related to GI events. Similar and low proportions of subjects (6–17 %) were unable to maintain their usual diet in both treatment sequences, with no notable difference between miglustat + *S. boulardii* and miglustat + placebo.

In an exploratory subgroup analysis of 12 subjects with diarrhea days while on miglustat + placebo, the mean (SD) number of diarrhea days during treatment was greater with miglustat + placebo (3.7 [2.8] days) than with miglustat + *S. boulardii* (2.1 [3.9] days): paired mean (SD) treatment difference −1.6 (3.9) days (p = 0.0635 based on Wilcoxon paired signed rank test).

### Safety

Overall, treatment-emergent AEs were recorded in 30 (75 %) subjects during miglustat + *S. boulardii* treatment and in 33 (85 %) subjects during miglustat + placebo treatment. Most AEs affected the GI system, and were mild or moderate. The incidence of the GI AEs also appeared to be slightly higher with miglustat + placebo (82 %) than with miglustat + *S. boulardii* (73 %). In order of decreasing incidence the most common GI AEs were flatulence, abdominal distension and diarrhea (Table 4). No subjects reported SAEs, and no subjects discontinued therapy due to AEs. No clinically relevant changes from baseline were observed in hematology or blood chemistry parameters, vital signs, ECG results or physical examination.

**Table 4:** Treatment-emergent GI AEs occurring in >10 % of subjects (all-treated set)

Category	Miglustat + *S. boulardii*	Miglustat + placebo
	(*n* = 40)^†^	(*n* = 39)^‡^
SOC GI disorders, n (%)	29 (73)	32 (82)
Total numbers of GI AEs	136	178
Individual AEs *n* (%):		
Flatulence	25 (63)	26 (67)
Abdominal distention	22 (55)	24 (62)
Diarrhea	17 (43)	19 (49)
Abdominal pain	13 (33)	13 (33)
Eructation	11 (28)	13 (33)
Dyspepsia	10 (25)	13 (33)
Abdominal discomfort	22 (55)	13 (33)
Nausea	5 (13)	12 (31)

*GI* gastrointestinal, *SOC* system, organ, class

^†^Miglustat + *S. boulardii* n = 40 (21 in Period 1 + 19 in Period 2);

^‡^Miglustat + placebo n = 39 (21 in Period 1 + 18 in Period 2)

### Pharmacokinetics

Miglustat pharmacokinetic analyses were based on measurements performed in 730 blood samples from a total of 37 (88 %) evaluable subjects. There were no significant differences in miglustat C_max_, AUC_τ_, T_max_ between the miglustat + *S. boulardii* and miglustat + placebo treatment periods. Geometric means (coefficient of variation) were 2.5 (24.8) h and 2.6 (25.8) h, respectively, for T_max_, 902 (40) ng/mL and 825 (43) ng/mL, respectively, for C_max_, and 5184 (43) h˕ng/mL and 4727 (47) h˕ng/mL, respectively, for AUC_τ_.

## Discussion and conclusions

Clinical studies have demonstrated that miglustat is a useful oral therapeutic option for the treatment of GD1 patients unable or unwilling to undergo intravenous ERT, and is the only disease-specific therapy for children and adults with NP-C [2]. The effective control of GI disturbances during miglustat therapy is an important clinical goal in both diseases as GI tolerability can adversely affect patient quality of life and compliance with therapy, and might hamper the effectiveness of treatment in some patients.

GI disturbances during miglustat therapy vary in frequency and severity according to the amount of ingested carbohydrates and the residual ability to digest them – patients who eat fewer or no carbohydrates while on miglustat report fewer GI disturbances, but are more likely to experience clinically significant weight loss due to decreased dietary caloric intake [11, 23].

Previous data have also demonstrated that GI enzyme substitution can reduce GI disturbances due to acquired disaccharidase deficiency [24–26]. *S. boulardii* has been shown to provide significant increases in the specific and total activity of sucrase-isomaltase (+82 %), lactase (+77 %), and maltase-glucoamylase (+75 %) in the intestinal mucosa after 8 days of oral treatment with 250 mg doses four times daily (total daily dose 1000 mg) [28]. In addition, a single-center, open-label, crossover Phase I study examined the safety, tolerability, and pharmacokinetics of miglustat 1000 mg t.i.d. administered in combination with sucrose, and with Baker’s yeast sucrase (invertase) or water (control), in asymptomatic HIV-1 positive patients. The study showed that addition of active sucrase (invertase) from Baker’s yeast extract reduced the incidence of loose or watery stools (data on file at Actelion).

The rationale for the use of the yeast strain, *S. boulardii*, is to restore the capacity of the human gut to digest sucrose. *S. boulardii,* given as a nutritional supplement while miglustat is co-administered, provides an exogenous sucrase (invertase) that hydrolyzes sucrose through a different mechanism from that of human sucrase [17]. Co-administration of *S. boulardii* would have the advantage of decreasing the need for dietary restriction or low-carbohydrate diets, and improve GI tolerability, comfort and compliance with miglustat therapy.

This is the first study to characterize GI AEs during miglustat therapy, in particular diarrhea, according to strictly defined and established clinical criteria, and during the first 2 weeks of miglustat therapy. The primary objective of this study, to assess whether co-administration of *S. boulardii* decreases the total number of diarrhea days in healthy subjects receiving miglustat, was not met. The primary and secondary endpoints to assess this objective did not show statistically significant treatment differences between miglustat + *S. boulardii* and miglustat + placebo. The number of diarrhea days in healthy subjects receiving miglustat + placebo was lower than expected based on clinical trial and post-marketing experience [3, 4, 6, 12], and limited the ability to detect a treatment effect with *S. boulardii*. This lower-than-expected number of diarrhea days may be due to the stringent definition used for diarrhea in the primary study endpoint as opposed to the definition of diarrhea when reported as an AE in clinical trials and practice. The frequency of diarrhea events reported as treatment-emergent AEs in this 2-week study (43 % with miglustat + *S. boulardii* and 49 % with miglustat + placebo) was approximately half of that reported in previous long-term clinical studies with miglustat (77 % during the first 6 months of treatment or 83 % at any time during miglustat treatment) [3, 4, 6, 12].

It is notable that further *post hoc* analyses of data from the PP set, which excluded one subject who presented with diarrhea of unknown cause prior to miglustat initiation while on *S. boulardii* only and with highly outlying data, gave positive results on the primary study endpoint (based on Wilcoxon paired signed rank test). The significantly lower number of diarrhea days observed after exclusion of this single subject suggests that certain patients receiving miglustat therapy may benefit from co-administration of *S. boulardii*.

General safety findings were in line with the known safety/tolerability profile for miglustat [2, 12], and there was no indication of any new safety findings identified as being associated with the use of miglustat in combination with *S. boulardii* in this population of healthy subjects. There were no notable treatment differences in miglustat PK parameters between the miglustat + *S. boulardii* and miglustat + placebo groups, which supports the hypothesis that co-administration with *S. boulardii* does not alter the steady-state PK of miglustat.

In terms of study limitations, while it would be preferable to assess the concomitant use of miglustat with *S. boulardii* in patients with approved indications (i.e., GD1 and NP-C), in whom the incidence of GI AEs has been adequately verified, it should be borne in mind that GD1 and NP-C are rare conditions. Healthy subjects were selected for this study for practical considerations, given the limited feasibility of patient recruitment within these indicated orphan populations and the fact that GI disturbances during miglustat treatment to date have generally been similar across all populations [2, 12]. It was therefore considered appropriate to extrapolate findings in healthy subjects to those in patients with GD1 or NP-C.

In summary, the primary endpoint analysis in this study did not demonstrate statistically significant improvement in the GI tolerability of miglustat co-administered with *S. boulardii*. A *post-hoc* analysis of the primary endpoint, which excluded a subject judged as a clear outlier, showed a statistically significant result, suggesting that the GI tolerability of miglustat might be improved with the concomitant use of *S. boulardii*.
